# Analysis of cellular and soluble profiles in QuantiFERON nonconverters, converters, and reverters in the Gambia

**DOI:** 10.1002/iid3.269

**Published:** 2019-08-20

**Authors:** Lydia Medawar, Hatimi Mohd Tukiman, Georgetta Mbayo, Simon Donkor, Olumuyiwa Owolabi, Jayne S. Sutherland

**Affiliations:** ^1^ Vaccines and Immunity Theme MRC Unit The Gambia at London School of Hygiene and Tropical Medicine Fajara Gambia; ^2^ Department of Biomedical Sciences University of Manchester Manchester UK; ^3^ Department of Infectious and Tropical Diseases London School of Hygiene and Tropical Medicine London UK

**Keywords:** cytokines, inflammation, QFT conversion, QFT reversion, Th17 cells, tuberculosis

## Abstract

**Background:**

Tuberculosis (TB) is the leading cause of death from a single infectious agent worldwide. The immune system is capable of clearing the pathogen before establishment of latent infection but the mechanisms for this are not yet understood.

**Methods:**

This study analysed highly exposed household contacts (HHC) of TB index cases who were categorised according to QuantiFERON (QFT) results at recruitment and 6 months. Seventeen (17) QFT nonconverters, 14 QFT converters, 18 QFT reverters and 18 latent TB infection (LTBI) were analysed. Supernatants generated following QFT stimulation at both time‐points were analysed using a 64‐plex cytokine array. Flow cytometry was performed on QFT converters and nonconverters at baseline only.

**Results:**

Interleukin‐2 (IL‐2), IL‐5, IL‐13, APRIL, IL‐17A, IP‐10, MIP‐1ß, sIL‐6rb, OPN, and sTNFR2 were all significantly higher in the QFT converters compared with nonconverters at baseline. Levels of interferon‐α2 (IFN‐α2) and IL‐2 were significantly lower in QFT reverters compared with nonconverters at baseline. Analysis of Ag‐specific IL‐2 levels resulted in an area under the curve (AUC) of 0.93 (95% confidence interval [CI], 0.84‐1.00) for QFT converters compared to nonconverters and an AUC of 0.80 (0.65‐0.95) for QFT reverters compared with LTBI. Purified protein derivative (PPD)‐specific CD4 + CD26 + IFN‐γ + cells were significantly increased (*P* = .0007) in QFT nonconverters compared with QFT converters at baseline.

**Conclusions:**

Our results provide insight into the underlying mechanisms of resistance to sustained *Mycobacterium tuberculosis* infection.

## 1 INTRODUCTION

Tuberculosis (TB) is the leading cause of death from a single infectious agent, and the ninth leading cause of death worldwide. In 2017, TB caused an estimated 1.6 million deaths, with 10.0 million new cases.^1^ Despite its prevalence there is a major gap in knowledge as to what constitutes natural protective immunity to TB, which precludes development of optimal vaccines. Whilst a vaccine is available (Bacillus Calmette–Guérin [BCG]), protection is highly variable and depends on age, setting and study quality.^2^, ^3^ Several pipeline vaccines are in development with the most successful, M72/AS01_E_, recently shown to provide 50% protection against adult pTB.^4^ However, the mechanism underlying this is unclear.^5^ Analysis of individuals who appear to “resist” development of latent TB infection (LTBI) and those who can actively clear infection would provide novel insights into natural protective immunity to Mtb.^6^

Evidence for natural protection from Mtb infection comes from the consistent finding that a proportion of highly *Mycobacterium tuberculosis* (Mtb)‐exposed individuals do not acquire Mtb infection.^7^, ^8^ This is best illustrated by the Lübeck disaster where ~20% of infants vaccinated with Mtb‐contaminated BCG did not get infected.^9^ In addition, studies of sailors in long‐term confinement with a TB patient showed a surprisingly low level of infection suggesting they were naturally resistant to Mtb infection.^10^ Importantly, longitudinal studies of these “resisters” confirm a much lower rate of progression to TB disease than those with LTBI.^11^ However, there are limitations in our interpretation of these cohorts based solely on IFN‐γ production with a recent paper highlighting potential IFN‐γ‐independent immune markers of *Mycobacterium tuberculosis* exposure.^12^ Thus, an expanded definition of the host response to Mtb exposure is needed.^12^

Sterilizing immunity is rarely achieved in animal and human Mtb infections. However, TST reversion was evident in 22% of guinea pigs exposed for up to 5 months to TB patients and this correlated with clearance of an established infection.^13^ Tuberculin skin test (TST) and QuantiFERON (QFT; a commercially available interferon (IFN)‐gamma release assay IGRA test) reversion are also evident in humans.^14^, ^15^ In a recent study BCG revaccination of adolescents had 45.4% efficacy against sustained Mtb infection. It was proposed that this reversion was due to a combination of innate and adaptive responses that generated enhanced control or, critically, clearance of bacteria^15^ but the underlying mechanisms are yet to be defined.

The cocktail of cytokines and chemokines produced by the body following Mtb exposure are crucial to the outcome of infection. IFN‐γ is a classical proinflammatory cytokine which is first induced by phagocytes in the innate immune system due to recognition of Mtb with pattern recognition receptors (PRRs) and subsequently by activated T cells.^16^ It has been used to define infection status in exposed household contacts following stimulation with Mtb‐specific antigens, as seen with the QFT assay.^14^ However, it is the balance between pro‐ and anti‐inflammatory mediators together that determines the outcome of the immune response to Mtb. Therefore this study aimed to analyse individuals who remained uninfected despite high exposure and those who cleared an active infection before establishment of sustained (ie, latent) Mtb infection to define the underlying immune mediators.

## 2 METHODS

### 2.1 Subject recruitment

This study was nested within a longitudinal cohort study of consecutively recruited household contacts at MRCG. Following identification of GeneXpert positive index TB patients, their exposed household contacts were assessed for signs and symptoms of TB. For asymptomatic individuals, the most highly exposed (ie, sleeping in the same room as the index TB patient) were included in this study after written informed consent. QFT‐GIT was performed at baseline and 6 months to determine Mtb infection status: QFT nonconverters = two negative readings; LTBI = 2 positive readings, QFT converters = negative at baseline and positive at 6 months and QFT reverters = positive at baseline and negative at 6 months. To avoid the “grey zone,” negative QFT was defined as less than or equal to 0.2 IU/mL and a positive QFT was defined as greater than or equal to 0.7 IU/mL. QFT supernatants from NIL, Mitogen and antigen tubes were stored and used for subsequent multiplex cytokine assays. For flow cytometry analysis, a heparinised venous blood sample was taken for PBMC separation and storage at baseline. This study was approved by the MRCG and Gambia government joint ethics committee.

### 2.2 Multiplex cytokine assays

Multiplex human cytokine assays were performed using Bio‐Plex Pro Human Cytokine 27‐plex kit (IL‐1β, IL‐1ra, IL‐2, IL‐4, IL‐5, IL‐6, IL‐7, IL‐8, IL‐9, IL‐10, IL‐12p70, IL‐13, IL‐15, IL‐17, basic FGF, eotaxin, G‐CSF, GM‐CSF, IFN‐γ, IP‐10, MCP‐1, MIP‐1α, MIP‐1β, PDGF‐BB, RANTES, TNF‐α, VEGF) and Bio‐plex Pro 37‐plex Human Inflammation Panel I (APRIL/TNFSF13, BAFF/TNFSF13B, sCD30/TNFRSF8, sCD163, Chitinase‐3‐like 1, gp130/sIL‐6Rβ, IFN‐α2, IFN‐β, IFN‐γ, IL‐2, sIL‐6Rα, IL‐8, IL‐10, IL‐11, IL‐12(p40), IL‐12(p70), IL‐19, IL‐20, IL‐22, IL‐26, IL‐27(p28), IL‐28A/IFN‐λ2, IL‐29/IFN‐λ1, IL‐32, IL‐34, IL‐35, LIGHT/TNFSF14, MMP‐1, MMP‐2, MMP‐3, Osteocalcin, Osteopontin, Pentraxin‐3, sTNF‐R1, sTNF‐R2, TSLP, TWEAK/TNFSF12 (BioRad, Belgium) according to the manufacturer's instructions. Briefly, lyophilised standards and controls were reconstituted in standard diluent. Coupled beads were diluted in assay buffer and 50 µL added to each well of a flat‐bottomed 96‐well plate. Fifty microliters of either standards, samples, blanks or controls was added to each well followed by an hour‐long incubation on an automated Microplate shaker at room temperature (RT). The plate was then washed three times in wash buffer and 25 µL of diluted detection antibodies were added to each well. Plates were wrapped in foil and shaken for 30 minutes at 400_gmax_. Plates were then washed three times and 50 µL of diluted SA‐PE was added to each well. The plate was wrapped in foil and shaken for 10 minutes at 400_gmax_. The plate was then washed three times, beads resuspended in 125 µL assay buffer, shaken for 30 seconds at 400_gmax_ and read using a Magpix multiplex plate reader (BioRad, Belgium).

### 2.3 Flow cytometry

#### 2.3.1 Antigen stimulation

Cryopreserved PBMC from QFT converters and nonconverters at baseline were thawed and rested for 6 hours at 37°C, 5% CO_2_ in RPMI + 10% FCS (RP10) + 0.1% Benzonase. Following resting cells were counted and 0.5 million viable cells stimulated with RP10 only (NIL) or PMA (cell stimulation cocktail, eBioscience). The following day PMA‐stimulated cells were restimulated overnight with specific antigens: ESAT‐6/CFP‐10 fusion protein (EC; kindly provided by Prof. T.H.M. Ottenhoff), PPD (both at a final concentration of 10 μg/mL) or RP10.

#### 2.3.2 Antibody staining

Following incubation, cells were washed in 1 mL fluorescence‐activated cell sorting (FACS) buffer (PBS/FCS/Az) at 600_gmax_, supernatant removed and incubated with live‐dead cascade yellow (Invitrogen, UK) for 10 minutes, RT. After washing, cells were stained with a surface cocktail consisting of anti‐CD3‐BV421, anti‐CD8‐eFluor 780, and anti‐CD26‐FITC (Biolegend, UK) and incubated for 15 minutes at 4°C. Following washing, cells were fixed (Fix/Perm buffer, BD) for 15 minutes at 4°C, permeabilised for 20 minutes at RT in the dark (Perm/Wash buffer, BD) and incubated with intracellular cytokines: anti‐IL‐17‐PE, anti‐TNF‐α‐PE‐Cy7, and anti‐IFN‐γ‐Alexa Fluor 700 (all from Becton Dickinson) for 30 minutes at RT in the dark. After a final wash, cells were resuspended in 300 μL of FACS buffer and acquired using a BD Fortessa.

### 2.4 Data analysis

Multiplex assay results were exported from Bio‐plex Manager Software version 6.1 (Bio‐Rad, Belgium) to Microsoft Excel. Cytokine concentrations outside the measurable range, as indicated by the Bio‐plex software, were set at either half the bottom standard concentration or double the top standard concentration, for those out of range below and out of range above, respectively. Cytokine responses of the unstimulated (NIL) samples were subtracted from the Ag and Mit data to generate Ag‐NIL and Mit‐NIL final results. Data were analysed with GraphPad Prism 7 (Software MacKiev). Mann‐Whitney or the Kruskal‐Wallis test (with Dunn's posttest comparison) was used to determine significant differences between the groups. Only *P* ≤ .035 were considered statistically significant, to account for False Discovery Rate (FDR). For flow cytometry analysis, raw FCS files were analysed using FlowJo v.10.4.2 (Becton Dickinson) with combination gates for Boolean cytokine analysis. Results for PMA‐only stimulated samples were subtracted from PMA + EC and PMA + PPD stimulated samples to generate EC and PPD specific responses. Differential cytokine responses between converters and nonconverters was analysed using PESTLE and SPICE.^17^

## 3 RESULTS

### 3.1 Characteristics of study participants

Seventeen (17) QFT nonconverters, 14 QFT converters, 18 QFT reverters, and 18 LTBI were included in the final multiplex cytokine analyses (Table 1). There were no significant differences in age between the groups with the median [interquartile range (IQR)] of 22 [18 to 28], 30 [25 to 60], 23 [19 to 35] and 31 [27 to 44], respectively. However, the LTBI group had a lower % of males compared with the other three groups (22% compared with 53% for TBR, 50% for QFTC and 39% for QFTR). The median [IQR] QFT Ag‐NIL IFN‐γ levels were 0[0‐0] and 0[0‐0.1] at baseline and 6 months for the QFT nonconverter group; 3.2 [0.6 to 10] and 5.6 [2 to 10] IU/mL at 0 and 6 months, respectively, for the LTBI group; 0[0‐0.1] and 1.6 [0.9 to 10] at 0 and 6 months, respectively, for the QFT converter group and 6.3 [2.5 to 10] and 0[0‐0.3] at 0 and 6 months, respectively, for the QFT reverter group (Table 1). Fifty‐nine percent of nonconverters and 71% of converters were BCG vaccinated (*P* = .70; relative risk 1.38). For flow cytometry analysis a subset of 10 QFT converters and 10 nonconverters were analysed. There were no differences in age or sex between the groups (Table 1).

**Table 1:** Study participants

Multiplex assay	Nonconverter	Converter	Reverter	LTBI
Multiplex assay, (n)	17	14	18	18
Age, median [IQR]	22 [18‐28]	30 [25‐60]	23 [19‐35]	31 [27‐44]
Males, n (%)	9 (53)	7 (50)	7 (39)	4 (22)
Baseline QFT, median [IQR]	0 [0‐0]	0 [0‐0.1]	6.3 [2.5‐10]	3.2 [0.6‐10]
6 month QFT, median [IQR]	0 [0‐0.1]	1.6 [0.9‐10]	0 [0‐0.3]	5.6 [2‐10]
BCG vaccinated n (%)	10 (59)	10 (71)	7 (39)	13 (72)
Flow cytometry, (n)	10	10	n/a	n/a
Age, median [IQR]	27 [21‐35]	32 [24‐63]	n/a	n/a
Males, n (%)	6 (60)	8 (80)	n/a	n/a

Abbreviations: LTBI, latent tuberculosis infection; IQR, interquartile range; QFT, QuantiFERON.

### 3.2 Inflammatory profiles in QFT converters versus nonconverters

**Figure 1:**
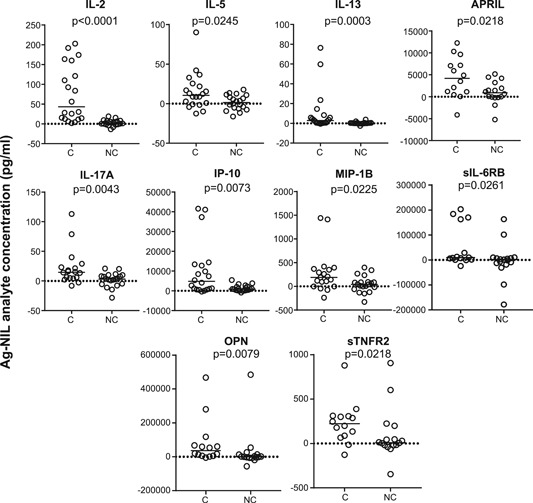
Analysis of Ag‐NIL supernatants in QFT converters (C) and nonconverters (NC). Whole blood was stimulated overnight with QFT Ag or NIL tubes. Luminex analysis was performed. Background was subtracted (Ag‐NIL) and data were analysed using the Mann‐Whitney *U* test. Line indicates median. *P* values are indicated. IL, interleukin; QFT, QuantiFERON

Analysis of Ag‐NIL cytokine levels at baseline showed that QFT converters had significantly higher levels of IL‐2 (*P* < .0001), IL‐5 (*P* = 0.0245), IL‐13 (*P* = .0003), APRIL (*P* = .0218), IL‐17A (*P* = .0043), IP‐10 (*P* = .0073), MIP‐1β (*P* = .0225), sIL‐6RB (*P* = .0261), Osteopontin (OPN) (*P* = .0079), and sTNFR2 (*P* = .0218) than the nonconverter group (Figure 1). Interestingly, the only analytes that showed a difference in unstimulated samples were CHI3L1 and IL‐9 which were both significantly higher in the converters compared with nonconverters at baseline (*P* = .0209 and *P* = .0278, respectively; data not shown). No differences in mitogen responses were seen between the groups at baseline (data not shown).

### 3.3 Inflammatory profiles in LTBI vs QFT reverters

**Figure 2:**
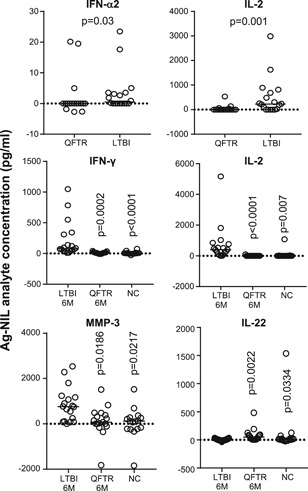
Analysis of Ag‐NIL supernatants in QFT Reverters (QFTR) vs LTBI. Whole blood was stimulated overnight with QFT Ag or NIL tubes. Luminex analysis was performed. Background was subtracted (Ag‐NIL) and data were analysed using the Mann‐Whitney *U* test. Line indicates median. *P* values are indicated. IL, interleukin; LTBI, latent TB infection; NC, nonconverters; QFT, QuantiFERON

Individuals with sustained infection (LTBI) vs QFT reverters were only analysed using the 37‐plex inflammation panel kit. In Ag‐NIL samples, the concentration of IFN‐γ, IL‐2, and IL‐22 were all significantly lower by 6 months compared with baseline in QFT reverters (*P* = .0002; *P* = .0039 and *P* = .0039, respectively) (data not shown). There was also a significantly lower level of IFN‐α2 and IL‐2 in QFT reverters compared with LTBI at baseline despite no difference in their QFT (IFN‐γ) levels (*P* = .03 and *P* = .001, respectively; Figure 2). Interestingly there were no differences between LTBI and active TB at baseline (data not shown). At the 6‐month time‐point (ie, post‐reversion), LTBI had significantly higher levels of IFN‐γ (*P* = .0002), IL‐2 (*P* < .0001), MMP‐3 (*P* = .0186) and IL‐22 (*P* = .0022) compared with QFT reverters and significantly higher levels compared with QFT nonconverters at baseline (*P* < .0001, *P* = .0007, *P* = .0217 and *P* = .0334, respectively; Figure 2). QFT reverters at 6 months had comparable levels of all analytes to QFT nonconverters supporting active clearance of the Mtb infection (Figure 2).

### 3.4 Receiver operated curve analysis

**Figure 3:**
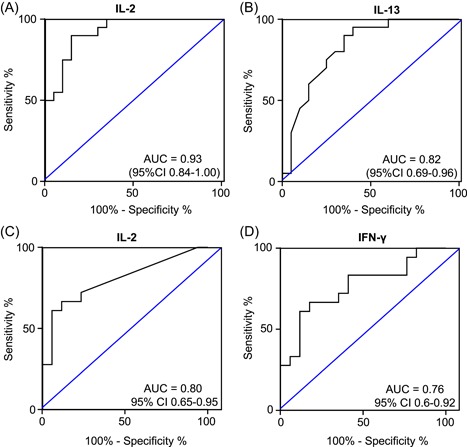
Receiver operated curve (ROC) analysis of Ag‐NIL supernatants. Nonconverters vs converters (A, IL‐2; B, IL‐13). QFT reverters vs LTBI (C, IL‐2; D, IFN‐γ). Whole blood was stimulated overnight with QFT Ag or NIL tubes. Luminex analysis was performed and background was subtracted (Ag‐NIL). Data were analysed using a ROC. Area under the curve (AUC) with 95% confidence intervals are indicated. IL, interleukin; IFN‐γ, interferon‐γ; LTBI, latent TB infection; QFT, QuantiFERON

Receiver operated curve (ROC) analysis was performed for QFT nonconverters compared with converters (Figure 3A and 3B) and for QFT reverters compared with LTBI (Figure 3C and 3D). For QFT converters and nonconverters, analysis of IL‐2 levels in Ag‐NIL supernatants at baseline gave an area under the curve (AUC) of 0.93 (95% confidence interval [CI], 0.84‐1.00) with a sensitivity of 90 (68‐99) % and specificity of 85 (62‐97) % at a cut‐off of 10.5 pg/mL (Figure 3A). The next best analyte for discrimination between QFT converters and nonconverters was IL‐13 with an AUC of 0.82 (95% CI, 0.69‐0.96) (Figure 3B). For LTBI vs QFT reverters, analysis of IL‐2 levels in Ag‐NIL supernatants at baseline resulted in an AUC of 0.80 (95% CI, 0.65‐0.95), sensitivity of 61 (36‐83) % and specificity of 94 (79‐100) % (Figure 3C) while IFN‐γ alone resulted in an AUC of 0.76 (95% CI, 0.6‐0.92) (Figure 3D).

### 3.5 Cellular immune profiles in QFT nonconverters

**Figure 4:**
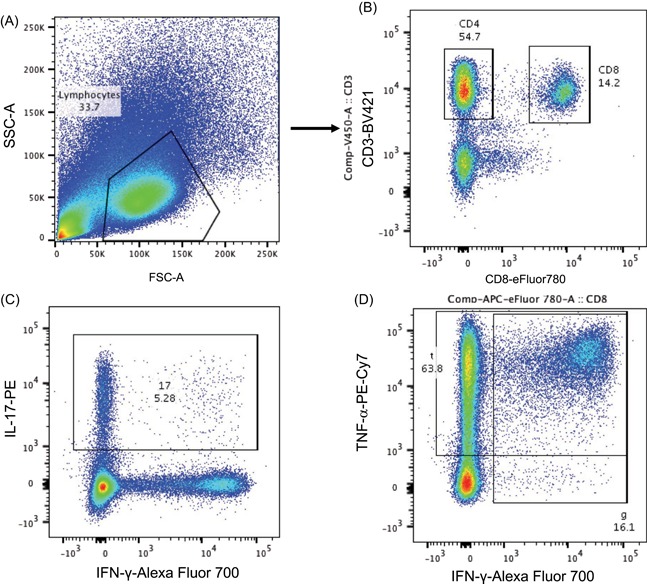
Gating strategy for analysis of polyfunctional CD4+ and CD8+ T cells. A, Lymphocytes were gated using FSC and SSC. B, From this, CD4+ and CD8+ T cells were gated using CD3 vs CD8 (CD3+ CD8+, CD8+ T cells; CD3+ CD8−, CD4+ T cells). Both CD4+ and CD8+ T cells were then analysed using Boolean gating for IL‐17, IFN‐γ (C) and TNF‐α (D). IL, interleukin; IFN‐γ, interferon‐γ; LTBI, latent TB infection; QFT, QuantiFERON

**Figure 5:**
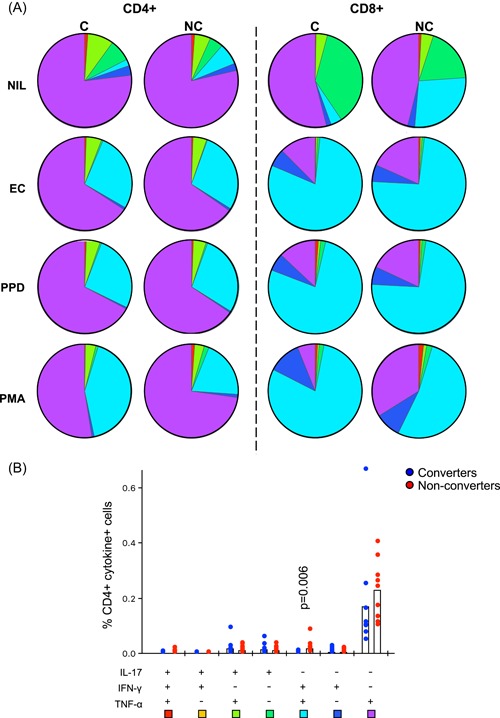
Polyfunctional T cell responses in QFT converters compared with nonconverters. A, Pie graphs indicating polyfunctionality of CD4+ T cells (left) and CD8+ T cells (right) responses to all stimulations used (NIL, EC‐PMA, PPD‐PMA, PMA alone). B, Graphical analysis of polyfunctional responses to NIL (unstimulated) samples in QFT converters (blue) compared with nonconverters (red). Data were analysed using the Mann‐Whitney *U* test. Colours correspond with pie section. QFT, QuantiFERON

Total CD4+, CD8+, and Th17 responses to Mtb antigens in QFT converters and nonconverters were analysed by flow cytometry to determine the role of adaptive immunity in Gambian QFT nonconverters. IFN‐γ, tumor necrosis factor (TNF‐α), and IL‐17 production was analysed using Boolean gating strategies and combinations of cytokines assessed for total CD4+, CD8+, and Th17 cells (Figure 4). When total CD4+ and CD8+ T cells were analysed, no difference in mono‐ or polyfunctional cytokine levels was seen for any stimulation (Figure 5A, pies). CD8+ T cells were predominantly IFN‐γ + TNF‐α + (light blue) with a small proportion producing IL‐17 either alone (dark green) or in conjunction with TNF‐α (light green) (Figure 5A CD8 + pies). CD4+ T cells were predominantly TNF‐α+mono‐functional (purple) followed by IFN‐γ + TNF‐α + (light blue) and IL‐17 + TNF‐α + (light green). Very few were IFN‐γ monofunctional (dark blue) in contrast to CD8+ T cells. The unstimulated (NIL) samples showed a significantly higher proportion of CD4 + IFN‐γ + TNF‐α + IL‐17‐ cells in QFT nonconverters compared with QFT converters at baseline (*P* = .006; Figure 5B).

**Figure 6:**
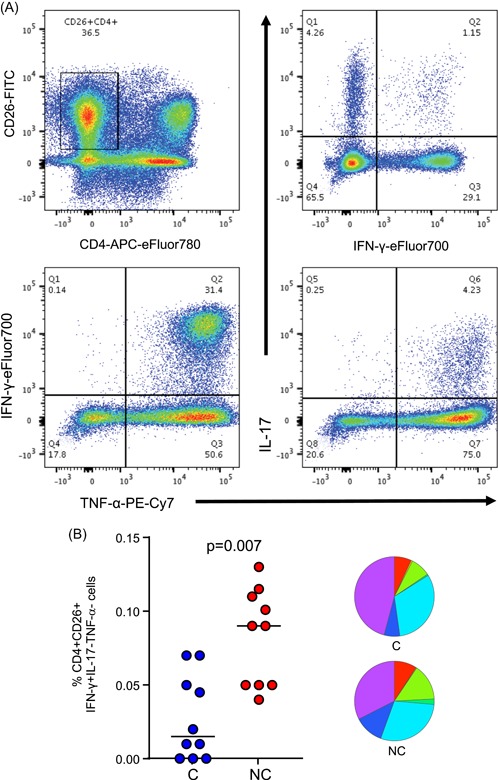
Analysis of Th17 polyfunctionality from QFT converters and nonconverters. A, Gating strategy for analysis of Th17 cells. CD26+ CD4+ cells were gated followed by Boolean gating of IL‐17+, IFN‐γ+, and TNF‐α+ cells. B, Analysis of IFN‐γ + IL‐17‐TNF‐α‐ CD26 + CD4 + T cells following PMA + PPD stimulation (PMA subtracted). Pies on right show all subsets analysed. Data were analysed using the Mann‐Whitney *U* test. Line indicates median. IL, interleukin; IFN‐γ, interferon‐γ; LTBI, latent TB infection; NC, nonconverters; QFT, QuantiFERON

We also analysed cytokine production from CD26+ CD4+ cells (Figure 6A). CD4+ CD26+ cells were highly polyfunctional with up to 2% of cells producing TNF‐α, IFN‐γ, and IL‐17 simultaneously following both EC and PPD stimulation (red pie section [only PPD shown]). Following PPD stimulation, QFT nonconverters had a significantly higher proportion of CD4 + CD26 + IFN‐γ + mono‐functional cells compared with QFT converters at baseline (*P* = .007; dark green pie; Figure 6B).

## 4 DISCUSSION

Analysis of individuals who are highly exposed to Mtb but never become latently infected and those who are able to actively clear the infection before development of sustained (latent) Mtb infection hold the key to understanding the mechanisms of early protection to Mtb. The aim of this study was to analyse cellular and soluble markers in highly exposed individuals who remain uninfected (QFT nonconverters) compared with those who become latently infected (QFT converters). We found several inflammatory mediators that were differentially expressed in QFT nonconverters compared with converters and QFT reverters compared with LTBI. In addition, QFT nonconverters had significantly higher levels of Mtb‐specific CD4 + IFN‐γ + TNF‐α + and Th1‐like CD26 + CD4 + cells (ie, IFN‐γ producing only^18^) cells at baseline compared with QFT converters.

In QFT converters, Mtb‐antigen‐specific levels of IL‐2 were significantly increased at baseline compared with nonconverters. In previous studies IL‐2 has been shown to enhance diagnosis of LTBI, particularly in those with borderline QFT results.^19^ In addition, IL‐2 levels are generally higher in LTBI compared with active TB patients.^20^ Our results showing elevation of IL‐2 before IFN‐γ conversion by QFT suggests IL‐2 is a likely marker of early infection leading to subsequent conversion. We also found that IL‐2 was significantly lower in QFT reverters at recruitment ie before reversion suggesting it is also decreased when the pathogen is being actively cleared and could be used as a biomarker for sterilizing TB. Importantly, at the follow‐up time‐point, levels of all analytes in the QFT reverter group were indistinguishable from the QFT nonconverters at baseline further suggesting they had actively cleared the infection. One limitation of our study was the lack of a third time‐point to assess the stability of the phenotypes analysed and this should be performed in future studies.

The second‐best marker for discrimination of QFT converters and nonconverters was IL‐13, which was also significantly higher at baseline in the QFT converters. IL‐13 is a Th2 cytokine closely related to IL‐4. It is thought that Th2 cytokines such as IL‐13 can cause inappropriate activation of macrophages and thus, undermine the Th1 response suitable for an intracellular pathogen like Mtb.^21^ We also found increased levels of IL‐17A, IP‐10 and MIP1β in converters compared with nonconverters. IL‐17A is associated with Th17 responses with a previous study from our laboratory showing contrasting findings (ie, increased IL‐17A in TBR).^22^ This difference may be due to the techniques used to classify the groups. In the initial study, an in‐house enzyme‐linked immunosorbent assay (ELISA) was used with only ESAT‐6/CFP‐10 peptides, whereas the present study used commercial QFT Gold‐In Tube, which includes additional peptides of TB7.7. The previous study also shohwed reduction in cellular IL‐17 production in nonconverters compared with converters following mitogen stimulation. We thus wanted to test whether this was also true for antigen‐specific responses. Interestingly we found no difference in the total proportion of Th17 cells, nor in the antigen‐specific production of IL‐17. However, we did see a significantly higher proportion of PPD‐specific Th‐1 like Th17 (CD26 + CD4 + ) cells in QFT nonconverters. QFT nonconverters also had significantly higher basal levels of CD4 + IFN‐γ + TNF‐α + cells. It is interesting to note that cellular IFN‐γ production appears to be elevated in QFT nonconverters despite a lack of QFT positivity, which is likely due to the antigens used for stimulation. QFT includes peptides to ESAT‐6 and CFP‐10, which we saw no difference in response to, but not to any broader Mtb antigens. The differential responses in our flow cytometry analysis were seen with PPD suggesting there could be some priming due to BCG. This should be tested in future studies. It was also a limitation that we did not analyse QFT reverters and did not include IL‐2 in our panel since this cytokine was discriminatory between the nonconverters and converters. Again, this should be addressed in future studies. It is interesting to note that the majority of differences we saw were in the adaptive immune system, which supports recent findings in a Ugandan cohort of Mtb “resisters.”^12^ However, another recent study from Indonesia showed stronger innate responses to heterologous stimuli in IGRA nonconverters.^23^ It's likely that different host and pathogen genetics will play a role but the cohort in Indonesia also included children which may have a bias to innate cell responses. Similarly, this may explain why we saw no influence of BCG on prevention of infection in our cohort.

In our study, participants were all HIV‐negative and those with QFT results in the grey zone were excluded. Thus, we have tried to control for spontaneous conversion/reversion. However, it is imperative that future studies include at least one more time‐point to ensure stability of both phenotypes. The fact that values for all cytokines reverted to levels seen in our nonconverters at baseline suggests that our findings are accurate, but inclusion of a further time‐point together with TST to exclude any discordant readings, would enable us to have a robust phenotype, which was outside the scope of the present study. Indeed, one of the major reasons for investigating QFT responses in our Gambian cohort is due to the lack of TST availability in‐country. It would also be important to assess responses at the site of infection through bronchoalveolar lavage (BAL)^24^ or PET/CT scanning^25^; neither of which are currently possible in The Gambia. Finally, it will be important to determine the cellular source of the soluble markers we have identified, particularly IL‐2. In addition, caution must be taken in regard to the definition of infection by IFN‐γ production alone as described in a recent paper by Lu et al.^11^

In conclusion, our results provide biological insight into the underlying mechanisms of resistance/active clearance of Mtb infection. Importantly, IL‐2 was shown to be a marker of both early conversion and early reversion while Th1‐like Th17 cells are potentially actively involved in early protective immunity to Mtb. Further studies should assess these factors in larger, ethnically diverse cohorts with further follow‐up time‐points together with assessment of the lung microenvironment.

## DATA ACCESSIBILITY

The data that support the findings of this study are available from the corresponding author upon reasonable request
